# Low-dose protocol of the spiral CT in orthodontics: comparative evaluation of entrance skin dose with traditional X-ray techniques

**DOI:** 10.1186/2196-1042-14-24

**Published:** 2013-09-10

**Authors:** Giancarlo Cordasco, Marco Portelli, Angela Militi, Riccardo Nucera, Antonino Lo Giudice, Elda Gatto, Alessandra Lucchese

**Affiliations:** Department of Orthodontics, University of Messina, Via Consolare Valeria, Messina, 98100 Italy; Department of Dentistry, Faculty of Medicine, San Raffaele Hospital, Vita-Salute San Raffaele University, Via Olgettina, Milano, 20132 Italy

## Abstract

**Background:**

The aim of this study was to evaluate the amount of radiation doses absorbed by soft tissues (entrance skin dose) with a low-dose spiral computed tomography (CT) protocol compared to conventional X-ray techniques commonly used in orthodontics.

**Methods:**

The amount of skin dose has been evaluated using a tissue-equivalent head-neck radiotherapy humanoid phantom with thermoluminescent dosimeters placed at the level of eye lens, parotid glands, and thyroid glands. CT images have been taken using a Sensation 16 Siemens CT scan and a low-dose protocol (15 mAs, 1 pitch, 2.5 mGy (CTDIvol), 80 kV, 1-mm slice thickness).

**Results:**

The difference in image quality between traditional X-ray techniques and low-dose spiral CT was statistically significant (*P* < 0.05). The difference in mean absorbed dose instead was not statistically significant.

**Conclusions:**

Our protocol allows a more accurate orthodontic diagnosis without an increase of radiological risk for the patients in comparison to traditional X-ray techniques.

## Background

Orthodontic diagnosis is primarily based on a morphological and quantitative description of cranial structures in sagittal, vertical, and transversal planes [1]. The exams traditionally used for both diagnosis and follow-up comprehend panoramic radiograph (PR), postero-anterior cephalogram (PAC), and lateral cephalogram (LC). The LC is a technique to transport human craniofacial structure into a measurable geometric scheme to evaluate the morphology and growth of craniofacial skeleton on sagittal and vertical planes [2]. PAC is often used to evaluate anomalies in the maxilla and in the mandible on the transverse plane including crossbite with ensuing functional or structural deviations of jaws. These conventional exams, however, present some limitations, related to their bi-dimensionality, to the superimposition of different anatomical structures on the same plane and to the radiographic projection, which is influenced by the patient's head position [3]. Studies demonstrated that there is no evidence about the reproducibility of landmark identification on PAC; limited evidence is available about random errors in the localization of landmarks on the PAC [4]. Orthodontists have long been interested in developing instruments that could allow them to obtain measurements in three dimensions with relative ease [5–8]. The development and introduction in medical practice of the computed tomography (CT) by Sir Godfrey Hounsfield, in 1971, represented an important contribution to the orthodontic radiological diagnosis, overcoming the limits of the previous methods.

The earliest application of CT in orthodontics goes back to 1979, when Montgomery et al. [9] investigated the accuracy of CT-based volume measurements of the nasal airway. In 1982, Timms et al. [10] used CT to examine the basal bone changes associated to rapid maxillary expansion.

Since the first prototypes, there has been a gradual evolution of CT scanner, which differs from each other in the organization of the individual part of the device and the physical motion of the beam in capturing the data. The latest generation scanners permit to reduce the movement and scatter artifacts [11]. CT apparatus X-ray source is a high-output rotating anode generator with a fan-shaped X-ray beam; the data are then recorded on a solid-state image detector arranged in a 360° array around the patient. Medical CT devices image patients in a series of axial plane slices that are captured either as stacked slices or from a continuous spiral motion over the axial plane [12]. The CT software gives cross-sectional images of the examined body with high-quality image of anatomical structures, and the spiral CT allows short time of scanning, volumetric attainment, multiplanar reconstruction, three-dimensional (3D) reconstruction of the hard and soft tissues of the skull, and 3D cephalometry; the obtained measurements are reliable because the image has 1:1 dimension, without distortion. Nevertheless, CT has some drawbacks: horizontal positioning of the patient during record taking falsifies the position of the facial soft tissue. There is also a lack of detailed occlusion due to artifacts and higher radiation exposure than other craniofacial X-ray acquisition systems [13].

The latter one is an important limitation of spiral CT, and it has spurred intensive research efforts to overcome it, beginning in 1998 with Hassfeld et al., who showed that by reducing the current in a so-called low-dose scanning protocol, the dose could be decreased to 76% without any reduction in diagnostic sensitivity [14]. Similar results were obtained in the study performed by Rustemeyer et al., who used a low-dose CT protocol for osseointegrated dental implant treatment planning. In this study, the doses to salivary glands and eye lens were determined with a tissue-equivalent phantom, and the authors demonstrated that by decreasing the current from 165 to 35 mAs and using a pitch factor of 2, a considerable dose reduction without the loss of diagnostic information is achievable in dental CT [15].

Similar conclusions were reached in the study by Homolka et al. [16], where the authors calculated the effective dose for dental CT protocol. The results indicate that by reducing the tube current, an effective dose for a CT examination of the maxilla of 22 μSv can be achieved, which is comparable to the values of PR (26 μSv), while a CT scan of the mandible gives 123 μSv comparable to a full-mouth survey with intra-oral films (150 μSv). For standard CT scan protocols of the mandible, effective doses exceed 600 μSv. The authors concluded that low-dose protocols for dental CT should be considered whenever feasible, especially for pediatric patients.

The aim of the present study is to comparatively evaluate the amount of entrance skin doses, obtained with a low-dose spiral CT protocol and those related to conventional techniques (PR, PAC, and LC), commonly used for orthodontic diagnosis.

## Methods

**Figure 1:**
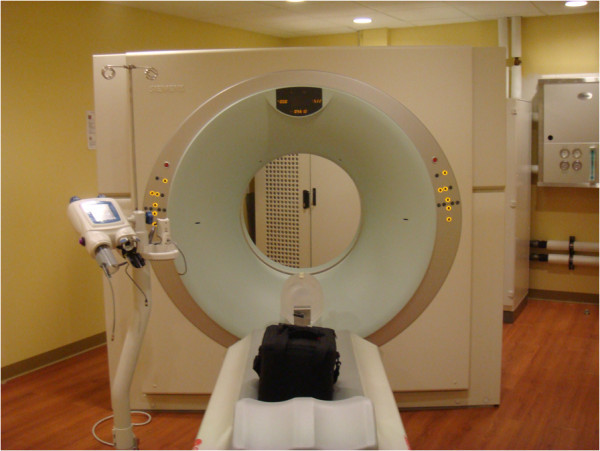
**CT scan (Somaton Sensation 16 Siemens®).**

**Figure 2:**
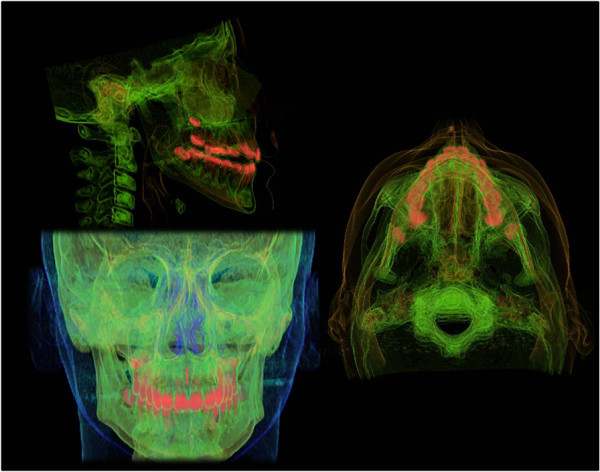
**CT images.**

CT exams were performed in the Department of Radiology of the University of Messina with a CT scanner (Somaton Sensation 16 Siemens®, Munich, Germany) equipped with a Dentascan reconstruction program, with 16 detector rows and the following characteristics: 420-ms rotation time, 30 lp/cm spatial resolution, and 5 mm/3 HU/19 mGy/180 mAs low-contrast detectability. A low-dose spiral CT protocol employed in our previous study was used [17]. The Ethical Committee of Messina University of Medical Sciences approved the ethical concerns of this study. This protocol has been obtained reducing the voltage and current and altering the pitch factor in order to decrease the radiation dose index for unit of volume (CTDIvol). The acquisition parameter values used were 15 mAs, 1 pitch, 2.5 mGy (CTDIvol), 80 kV, and 1-mm slice thickness. This protocol has been suggested as a low dose, because the acquisition parameter values used were the lowest ones allowed by the apparatus in order to perform the exam and, at the same time, they guaranteed a good image quality. The amount of radiation effectively absorbed by the soft tissue (skin dose) was measured using dosimeters placed at the level of eye lens, parotid glands, and thyroid glands. These anatomic regions have been selected for their high X-ray sensibility, related to their high mitotic index, and for their position close to the direct radiation area. The Siemens 16 CT Sensation machine was employed, using a tissue-equivalent head-neck radiotherapy humanoid phantom (Standard ATOM® Phantoms, CIRS, Norfolk, VA, USA) (Figures 1 and 2). Thermoluminescent dosimeters (TLD; Atomtex® TLD, Minsk, Republic of Belarus) were used; each TLD had a card with various slots where different filters could be fitted, and two sensors were positioned into them. The correct reading of the card (Rados-Dosacus-Tid Reader, Atomtex® TLD) and the software system that converts the LED dose signal were guaranteed by the localized light beam (doserad ii) with a sit commencer at the center; 20 μGy was the minimum legible dose, with 10% max per dose of uncertainty and <5% reproducibility. The doses were registered for five times; mean value and standard deviation were calculated. The volume of the examined area extended from the fronto-nasal suture to the caudal extremity of the mandible. For the conventional techniques, we used a digital apparatus (Proline XC, Planmeca, Helsinki, Finland) with the following pediatric acquisition parameter values:

- L and P-A projections → 8 mA, 66 kV, 1 and 2 s
- Panoramic → 8 mA, 66 kV, 15 s

The quality of the images was evaluated on the basis of information useful for orthodontic diagnosis and considering the level of tissue contrast, image sharpness, and overall subjective impression. For the assessment of image quality, during the first reading session, two readers, independent and experienced orthodontists, were asked to make the diagnosis. The criteria used to establish the level of quality for orthodontic diagnosis was the reliability and the reproducibility in the identification of the anatomical landmarks traditionally used for the analysis of both LC and PAC. In the sagittal plane, the identification of the following landmarks was evaluated:

- Point A (deepest point of the curve of the maxilla, between the anterior nasal spine and the dental alveolus)
- Point B (most posterior point in the concavity along the anterior border of the symphysis)
- Point sella (center of the pituitary fossa of the sphenoid bone)
- Point N (intersection of the inter-nasal suture with the nasofrontal suture in the midsagittal plane)
- Point gonion (most convex point along the inferior border of mandibular ramus)
- Point pogonion (most anterior point on the midsagittal symphysis)
- Point condilion (most posterior superior point of the condyle)
- Point SNA (tip of the anterior nasal spine)
- Point SNP (tip of the posterior nasal spine)

In the frontal plane, the identification of the following landmarks was evaluated:

- Zygomatic arch, right and left (center of the root of the zygomatic arch)
- Crista galli (most superior point of crista galli)
- Lateral wall of the nasal cavity, right and left
- Menton (most inferior point of the symphysis)
- Jugal process, right and left (intersection of zygomatic buttress and outline of the tuberosity)
- Antegonial notch, right and left (lateral inferior margin of the antegonial protuberance)

Intra- and inter-observer reproducibilities were evaluated both for CT, LC, and PAC. In order to perform the intra-examiner reproducibility, the orthodontists repeated the measurements after 7 days. Concordance between the two groups of measurements performed by the orthodontists on CT, LC, and PAC was evaluated to state the image quality useful for orthodontic diagnosis.

In a second reading session, the other two readers, independent and experienced radiologists, were asked to rate the overall image quality by considering tissue contrast, image sharpness, and overall subjective impression. Artifacts due to the patient's movements were rated as follows: no artifacts, few artifacts, moderate artifacts, and severe artifacts. Images were rated according to the following scores matched by a corresponding numerical score ranging from 1 to 5: poor, moderate, satisfactory, good, and excellent.

Written informed consent was obtained from the patient for publication of this report and any accompanying images.

### Statistical analysis

Results were compared using Wilcoxon’s (matched pairs) signed rank test to identify significant difference in terms of image quality and absorbed dose. The level of significance was set at *p* < 0.05. Descriptive statistics included mean and standard deviation (Table 1).

**Table 1:** **Absorbed doses (mGy)**

X-ray technique	Right eye lens		Left eye lens		Right parotid gland		Left parotid gland		Right thyroid gland		Left thyroid gland	
	Mean	SD	Mean	SD	Mean	SD	Mean	SD	Mean	SD	Mean	SD
LC	0.06	0.012	0.09	0.021	0.04	0.0098	0.3	0.052	0.06	0.013	0.07	0.017
PAC	0.28	0.04	0.31	0.056	0.03	0.0087	0.05	0.011	0.07	0.018	0.04	0.009
PR	0.07	0.016	0.08	0.019	0.52	0.14	0.49	0.12	0.08	0.039	0.07	0.0195
LC + PAC + PR	0.41	0.097	0.48	0.101	0.59	0.158	0.84	0.32	0.21	0.097	0.18	0.022
Low-dose CT	0.57	0.11	0.58	0.12	0.78	0.289	0.79	0.30	0.18	0.081	0.17	0.067

For each X-ray technique and organ, the doses have been registered five times. The mean value and the standard deviation (SD) are reported.

## Results

Table 1 shows the doses absorbed by different organs when performing the LC, PAC, PR, and low-dose CT. In the LC, the highest doses were absorbed by the left eye lens and the left parotid and thyroid glands and were 0.09, 0.3, and 0.07 mGy, respectively. In the PAC, the highest absorbed doses were observed in the left eye lens, left parotid gland, and right thyroid gland, being 0.31, 0.05, and 0.08 mGy, respectively. For PR, the highest doses were absorbed by the left eye lens, right parotid gland, and right thyroid gland and were 0.08, 0.52, and 0.08 mGy, respectively. With low-dose spiral CT, the highest absorbed doses were detected in the left eye lens, left parotid gland, and right thyroid gland (0.58, 0.79, and 0.18 mGy, respectively). Other data show that the highest radiation dose, both with conventional radiography (LC, PAC, and PR) and low-dose spiral CT, was absorbed by the left parotid gland (0.84 and 0.79 mGy, respectively), followed by the left eye lens (0.48 and 0.58 mGy, respectively) and the right thyroid gland (0.21 and 0.18 mGy, respectively). The doses were not subjected to the TLD signal fluctuation, and they were calculated subtracting the background TLD dose. In Table 2, the image quality values assigned by experienced radiologists and orthodontists are reported, together with the mean absorbed doses for the different X-ray techniques. Regarding the measurements performed by the orthodontists, the concordance between the two groups of measurements performed on CT image was quite high (0.986 for the intra-examiner calibration and 0.980 for the inter-examiner calibration) and, in all cases, was statistically significant (*P* < 0.001). Concordance between the two groups of measurements performed on LC and PAC was instead quite low (0.267 for the intra-examiner calibration and 0.273 for the inter-examiner calibration) and, in all cases, was not statistically significant (*P* > 0.05).

**Table 2:** **Image quality scores attributed and mean absorbed doses registered**

	Image quality	Mean absorbed dose (mGy)
LC	Satisfactory (3)	0.10
PAC	Moderate (2)	0.78
PR	Good (4)	0.21
LC + PAC + PR	Satisfactory (3)	0.45
Low-dose CT	Excellent (5)	0.51

In Table 3, the data related to the statistical analysis of image quality and mean absorbed doses are reported.

**Table 3:** **Wilcoxon's signed-rank test to identify significant difference in terms of image quality and absorbed dose**

	LC + PAC + PR	Low-dose CT	***P*** value
Image quality	Satisfactory (3)	Excellent (5)	<0.05
Mean absorbed dose (mGy)	0.45	0.51	NS

The level of significance was set at *P* < 0.05. NS, not statistically significant.

## Discussion

Radiation dose should be kept as low as reasonably achievable both for patient and operator as recommended by the American Dental Association's Council on Scientific Affairs. The hypothesis in modern radiation protection is that any dose of radiation has the potential to cause biological harm. The probability of long-term effects (stochastic effects) of radiation increases with the exposure dose, but the severity of potential consequential effects is not affected. In small children, the vulnerability to radiation is higher due to the large number of cell divisions occurring in this patient population [18]. CT scans are often performed using adult exposure parameters that do not consider the age and size of the patient, resulting in unnecessary high radiation exposures [19]. Radiography should be performed only when a patient's history and/or objective findings lead to the conclusion that further useful information might be warranted. If a radiograph is not expected to change diagnosis or treatment or add other useful information, it should not be taken [18]. This claim is supported also by an editorial published by Turpin regarding the radiographic guidelines proposed by the British Orthodontic Society [20].

The low-dose spiral CT protocol proposed in this study produced a mean absorbed dose similar to that related to conventional radiographic exams (LC + PAC + PR), with a difference of about 0.06 mGy. Intra- and inter-observer calibrations showed that CT images allow a more accurate and reliable identification of anatomical landmarks necessary for orthodontic diagnosis. Considering these results, it is possible to state that the protocol proposed in this study provides more accurate information than traditional X-ray techniques without a significant increase of radiological risk for the patients.

In our study, the left region of the examined organs absorbed the highest radiation dose, a finding that we ascribe to the location of these organs in the primary radiation field of the X-ray beam [21]. Our low-dose protocol entails a considerably lower radiation exposure than other low-dose protocols such as the one employed by Ekestubbe et al. [22], who used CT with a current of 40 to 280 mAs. The doses delivered with our low-dose settings are also lower than those described in other studies using spiral CT with low-dose parameters and with an anthropomorphic phantom, such as the study by Cohnen et al. [23], which compared radiation exposure using a dose-reduced CT protocol, with radiation doses ranging from 10.9 to 6.1 mGy.

The reduction of tube power is the most important measure to obtain a reduction of dose, and further technical modifications should be carried out. This is of utmost importance for the examination of children, where reducing the tube current, considering a smaller body diameter, would be sufficient for producing good images with a significantly lower amount of radiations [24].

Using this low-dose spiral CT protocols, clinicians could have the possibility to acquire high-quality images useful for a more accurate diagnosis, especially in the cases of difficult anatomical landmark identification [25]. The protocol proposed in this study could be applied not only in orthodontics but in any field of dentistry, becoming an important diagnostic instrument for different types of pathologies that may affect the craniofacial region.

By now, a new generation of computed tomography apparatus, the cone beam computed tomography (CBCT), has been developed. CBCT technology uses a cone-shaped X-ray beam with a special image intensifier and a solid-state sensor or an amorphous silicon plate for capturing the image, and reconstructions are performed in post-processing via algorithms [12]. CBCT is advocated because, in comparison to conventional CT, it offers the advantage of low radiation dose, short time of scan, low cost, natural shape of the soft tissue facial mask due to vertical scanning position, and reduced artifacts at the level of the occlusion. The drawback of CBCT is the limited reliability of post-processing algorithm reconstruction that can affect image reliability. Some recent dosimetric studies state that CBCT scans produce a smaller effective dose to patients if compared to multislice CT scans [26, 27]. A national guidance on CBCT has been also developed by Sedentex [28]; the conclusion of these guidelines is that the conventional approach adopted for common dental X-ray equipment is inappropriate or even unsafe for CBCT scans. For this reason, in order to provide an adequate level of radiation, protection is necessary to adopt some wariness. However, a systematic review of De Vos et al. [29] indicates a lack of evidence-based data on the radiation dose for CBCT imaging. Terminology and technical device properties and settings are not consistent in the literature. Because CBCT scanners have unique radiation geometry, no agreement has been reached yet on how CBCT dosimetry should be measured in terms of radiation detector setup in phantom and geometrical calculation. A minimal set of CBCT device-related parameter values for dedicated oral and maxillofacial region scanners is proposed as a guideline for future studies.

## Conclusions

Although the true cancer risk with low-dose radiation is debated, there is a consensus that the radiation dose for a particular imaging study should be minimized. Therefore, when it is determined that the potential benefits from the information obtained by spiral CT outweigh the risk of the radiation dose, technical settings should be adjusted to minimize the radiation dose.

Our protocol resulted in considerable reduced doses without any loss of diagnostic image quality, allowing a more accurate orthodontic diagnosis. This type of protocol should be used only in cases in which a more accurate radiographic evaluation is necessary, such as facial asymmetry, impacted teeth, orthognathic cases, etc. [30, 31]. In these cases, in fact, CT images provide more accurate information than traditional X-ray techniques without a significant increase of radiological risk for the patients.

## References

[1] Angle EH (1907). Treatment of Malocclusion of the Teeth.

[2] Moyers RE, Bookstein FL, Hunter WS, Moyers RE (1998). Analysis of the craniofacial skeleton: cephalometrics. Handbook of Orthodontics.

[3] Park SH, Yu HS, Kim KD, Lee KJ, Baik HS (2006). A proposal for a new analysis of craniofacial morphology by 3-dimensional computed tomography. Am J Orthod Dentofacial Orthop.

[4] Leonardi R, Annunziata A, Caltabiano M (2008). Landmark identification error in posteroanterior cephalometric radiography. Angle Orthod.

[5] Baumrind S, Moffltt FH, Curry S (1983). Three-dimensional X-ray stereometry from paired coplanar images: a progress report. Am J Orthod.

[6] Broadbent BH (1931). A new X-ray technique and its application to orthodontia. Angle Orthod.

[7] Grayson BH, McCarthy JG, Bookstein F (1983). Analysis of craniofacial asymmetry by multiplane cephalometry. Am J Orthod.

[8] Grayson BH, LaBatto FA, Kolber AB, McCarthy JG (1985). Basilar multiplane cephalometric analysis. Am J Orthod.

[9] Montgomery WM, Vig PS, Staab EV, Matteson SR (1979). Computed tomography: a three-dimensional study of the nasal airway. Am J Orthod.

[10] Timms DJ, Preston CB, Daly PF (1982). A computed tomographic assessment of maxillary movement induced by rapid expansion - a pilot study. Eur J Orthod.

[11] Kau CH, Richmond S, Palomo JM, Hans MG (2005). Three-dimensional cone beam computerized tomography in orthodontics. J Orthod.

[12] Mah J, Hatcher D (2004). Three-dimensional craniofacial imaging. Am J Orthod.

[13] Swennen GRJ, Schutyser F (2006). Three-dimensional cephalometry: spiral multi-slice vs cone-beam computed tomography. Am J Orthod.

[14] Hassfeld S, Streib S, Sahl H, Stratmann U, Fehrentz D, Zöller J (1998). Low-dose computerized tomography of the jaw bone in pre-implantation diagnosis. Limits of dose reduction and accuracy of distance measurements. Mund Kiefer Gesichtschir.

[15] Rustemeyer P, Streubühr U, Hohn HP, Rustemeyer R, Eich HT, John-Mikolajewski V, Müller RD (1999). Low-dosage dental CT. Rofo.

[16] Homolka P, Gahleitner A, Kudler H, Nowotny R (2001). A simple method for estimating effective dose in dental CT. Conversion factors and calculation examples for a clinical low dose protocol. Rofo.

[17] Matarese G, Portelli M, Mazza M, Militi A, Nucera R, Gatto E, Cordasco G (2006). Evaluation of skin dose in a low dose spiral CT protocol. Eur J Paed Dent.

[18] Espelid I, Mejàre I, Weerheijm K (2003). EAPD guidelines for use of radiographs in children. Eur J Paed Dent.

[19] Gelfand MJ, Lemen LC (2007). PET/CT and SPECT/CT dosimetry in children: the challenge to the pediatric imager. Semin Nucl Med.

[20] Turpin DL (2008). British Orthodontic Society revises guidelines for clinical radiography. Am J Orthod.

[21] Bou Serhal C, Jacobs R, Gijbels F, Bosmans H, Hermans R, Quirynen M, van Steenberghe D (2001). Absorbed doses from spiral CT and conventional spiral tomography: a phantom vs. cadaver study. Clin Oral Implants Res.

[22] Ekestubbe A, Gröndahl K, Ekholm S, Johansson PE, Gröndahl HG (1996). Low-dose tomographic techniques for dental implant planning. Int J Oral Maxillofac Implants.

[23] Cohnen M, Kemper J, Möbes O, Pawelzik J, Mödder U (2002). Radiation dose in dental radiology. Eur Radiol.

[24] Statkiewicz S (2010). Radiation Protection in Medical Radiography.

[25] Marsico E, Gatto E, Burrascano M, Matarese G, Cordasco G (2011). Effectiveness of orthodontic treatment with functional appliances on mandibular growth in the short term. Am J Orthod Dentofacial Orthop.

[26] Soumalainen A, Kiljunen T, Kaser Y, Peltola J, Kortesniemi N (2009). Dosimetry and image quality of four dental cone beam computed tomography scanners compared with multislice computed tomography scanners. Dentomaxillofac Radiol.

[27] Loubele M, Bogaerts R, Van Dijck E, Pauwels R, Vanheusden S, Suetens P, Marchal G, Sanderink G, Jacobs R (2009). Comparison between effective radiation dose of CBCT and MSCT scanners for dentomaxillofacial applications. Eur J Radiol.

[28] SEDENTEXCT (safety and efficacy of a new and emerging dental X-ray modality) [Internet] Manchester (Greater Manchester, UK): University of Manchester; 2013 – [cited 2009 July]. Available from:

[29] De Vos W, Casselman J, Swennen GRJ (2009). Cone-beam computerized tomography (CBCT) imaging of the oral and maxillofacial region: a systematic review of the literature. Int J Oral Maxillofac Surg.

[30] Lucchese A, Gherlone E, Portelli M, Bertossi D (2012). Tooth orthodontic movement after maxillofacial surgery. Eur J Inflamm.

[31] Lucchese A, Carinci F, Brunelli G (2012). Use of ferric-sulphate gel for bleeding control in surgical exposure of impacted canines. Eur J Inflamm.

